# Supplementary material for: Replication of a neuroimaging biomarker for striatal dysfunction in psychosis
Source: Res Sq. 2023 Aug 7:rs.3.rs-3185688. Preprint. [Version 1] doi: 10.21203/rs.3.rs-3185688/v1 (PMC10441472; doi:10.21203/rs.3.rs-3185688/v1)
Supplement: Supplement 1 [file NIHPPrs3185688v1-supplement-1.pdf]

Supplementary Files

This is a list of supplementary files associated with this preprint. Click to download.

- SupplementaryInformation.docx
